# Paroxysmal Nocturnal Hemoglobinuria (Pnh): Brain Mri Ischemic Lesions In Neurologically Asymtomatic Patients

**DOI:** 10.1038/s41598-017-18936-0

**Published:** 2018-01-11

**Authors:** Wilma Barcellini, Elisa Scola, Silvia Lanfranconi, Marika Grottaroli, Francesca Binda, Bruno Fattizzo, Anna Zaninoni, Gloria Valcamonica, Claudia Maria Cinnante, Carla Boschetti, Massimiliano Buoli, Carlo Alfredo Altamura, Nereo Bresolin, Fabio Triulzi, Alberto Zanella, Agostino Cortelezzi

**Affiliations:** 1 Hematology Unit, IRCCS Foundation Ca’ Granda Ospedale Maggiore Policlinico, Milan, Italy; 2 Neuroradiology Unit, IRCCS Foundation Ca’ Granda Ospedale Maggiore Policlinico, Milan, Italy; 3 Neurology Unit, IRCCS Foundation Ca’ Granda Ospedale Maggiore Policlinico, Milan, Italy; 4 Department of Psychiatry, IRCCS Foundation Ca’ Granda Ospedale Maggiore Policlinico, Milan, Italy; 5 Dino Ferrari Centre, Neuroscience Section, Department of Pathophysiology and Transplantation (DEPT), University of Milan, Milan, Italy; 6 University of Milan, Milan, Italy

## Abstract

This study investigated for the first time brain ischemic involvement in 19 consecutive neurologically asymptomatic PNH patients by non-enhanced cerebral MRI, and by intracranial arterial and venous angio-MRI. Eleven cases (58%, 7 aged <65) showed pathological findings: 9 white matter (WM) abnormalities related to chronic ischemic small vessel disease, 2 a focal abnormality >5 mm, and 5 cases a score >4 by the age-related white matter changes (ARWMC) scale. Compared with age and sex-matched controls (1:2 ratio), patients showed an increased frequency of periventricular WM vascular degeneration (32% versus 5.2%, p = 0.04) and of severe lesions (ARWMC scale score >4) (26% versus 2.6%, p = 0.05), and a higher overall ARWMC scale score (3.5 ± 1.07 versus 2.0 ± 0.8, mean ± SD, p < 0.0001). Notably, vascular abnormalities suspected for prior partial venous thrombosis, were observed in PNH cases only. MRI lesions were not related to blood counts, hemolytic markers, clone size, disease duration, and therapy with eculizumab. Neurological examination was unremarkable in all patients but one (Parkinson disease). Psychiatric assessment revealed a case of generalized anxiety disorder, 1 bipolar disorder type 2, and 1 adjustment disorder. In conclusion, brain MRI may be useful at diagnosis and during the course of the disease to explore subclinical neurological involvement.

## Introduction

Paroxysmal nocturnal hemoglobinuria (PNH) is a rare acquired stem cell disorder (incidence of 2–6 per million) characterized by hemolytic anemia, marrow failure and thrombosis. It is due to a mutation in the phosphatidylinositol glycan class A (PIG-A) gene, which results in a deficiency of glycosylphosphatidyl-inositol (GPI)-anchored proteins, including complement regulatory CD55 and CD59. In particular, erythrocytes belonging to a PNH clone are abnormally sensitive to complement activation (exacerbated by surgery, infection, inflammation, or pregnancy), resulting in chronic intravascular hemolysis^1^. Venous thrombosis, particularly in abdominal and in intracranial veins, is the leading cause of mortality in patients with PNH, accounting for 40–67% of deaths with known causes^2^. Several mechanisms, including prothrombotic microparticles, proinflammatory cytokines and complement factors, activated platelets, and defective fibrinolysis have been hypothesized to play a role; however, the precise bases of this thrombophilic state, sometimes occurring in a thrombocytopenic patient, are still unknown. In addition, high plasma levels of free hemoglobin and subsequent depletion of nitric oxide (NO), a regulator of smooth muscle tone, may contribute to microvascular thrombosis, accounting for symptoms such as dysphagia, abdominal pain, headache, and erectile dysfunction^1–4^. Altogether, subclinical microthrombi and hemolysis-associated NO scavenging result in organ damage and death: PNH patients have an increased risk of chronic kidney disease and pulmonary hypertension and a reduced 10-year survival rate (50% for patients diagnosed before 1970 and 75% in a more recent series)^4–7^. Today, eculizumab, a humanized monoclonal antibody that inhibits terminal complement activation, has dramatically changed the natural history of the disease, providing a 92% reduction in the risk of thromboembolism, along with an effective reduction of hemolysis and transfusion requirements, and with improvements in pulmonary hypertension and renal function^8–13^. Thrombotic events in PNH generally occur in unusual sites, such as hepatic, portal, mesenteric, splenic, and renal veins. Brain involvement is anecdotic in the literature^14–16^. The largest series reported^17^ described 15 PNH cases with cerebral venous thrombosis, mostly women, and younger than a control population without PNH; 3 cases had a concomitant splancnic thrombosis and 1 patient died. Few cases (about 9) of ischemic strokes are reported, that were fatal in about 1/3 of cases^18^. More recently, in a PNH case with mild left hemiparesis, a brain computed tomography scan showed multiple lacunar infarcts, and a magnetic resonance demonstrated several acute and chronic ischemic stroke areas^19^. Moreover, cerebral occlusive lesions involving various intracranial arteries have been reported in PNH as a result of Moya-Moya phenomenon^20,21^. However, no systematic studies have been reported in neurologically asymptomatic patients. The aim of this study was to investigate brain involvement in asymptomatic PNH patients, either or not in eculizumab treatment, by non-enhanced cerebral magnetic resonance imaging (MRI), and by intracranial arterial and venous angio-MRI, and to compare MRI findings with an age and sex-matched control group. Neuro-radiological patients’ findings were completed with a neuro-psychiatric evaluation, and correlated with clinical/hematologic features and therapy.

## Results

### Clinical and hematological characteristics of patients

The main clinical and hematological parameters of patients are shown in Table 1. Seventeen out of 19 patients were classical hemolytic (63% transfusion dependent before treatment with eculizumab and 1 patient also after), and 2 PNH in the context of aplastic anaemia (all transfusion-dependent until treatment with ATG-CyA). The majority of patients were female (M:F ratio of 0.46), and median age at diagnosis was 44 years, with a wide range (17–80). Asthenia and dyspnea on exertion were present in all patients and abdominal pain in 42% (N. 1, 2, 4, 6, 11, 12, 15, and 16). Four cases (N. 2, 6, 8 and 12) experienced a thrombotic event involving the sovrahepatic veins, the retinal arteria and the renal veins, respectively. Hemoglobin (Hb) values showed a great variability (median 9.6 g/dL, range 6.7–12.9) as well as LDH, with a median increase of 3.7-fold (range 1.2–16.3) over upper limit of normal (ULN). Seventy-three per cent of patients displayed a clone size greater than 50% GPI negative cells, and only 2 patients lower than 20%. Thrombotic events were not correlated with clone size, as they occurred in 3 cases with a clone size >50% but also in a patient with a clone size <50%. However, patients who had a thrombotic event displayed greater median LDH levels (7.2 ± 6.1 versus 4.6 ± 3.1 folds over ULN, mean ± SD), and median clone size (73.5 ± 26 versus 59.8 ± 23% of GPI negative cells, mean ± SD) than cases without thrombosis. As regards abdominal pain, median LDH levels were greater in symptomatic cases (6.8 ± 4.8 versus 3.8 ± 2.5 folds over ULN, mean ± SD), while the clone size was comparable in subjects with or without symptoms.

**Table 1:** Clinical and hematologic parameters of PNH patients.

**Patient N**.	**Year of diagnosis**	**PNH type**	**Data at diagnosis**					**Therapy with eculizumab, starting date**	**Data at last follow-up (2015/2016)**				
			**Age (yrs)/ gender**	**Hb (g/dL)**	**LDH (% ULN)**	**PMN (% GPI neg)**	Reticulocytes (*10 ^**3**^ **/mmc)**		**Age (yrs)**	**Hb (g/dL)**	**LDH (% ULN)**	**PMN (% GPI neg)**	Reticulocytes (*10 ^**3**^ **/mmc)**
1	1996	hemolytic	48/F	12,0	9,5	48	126	yes, 2005	68	11,9	1,2	33	80
2	1997	hemolytic	31/F	8,2	4,3	63	251	yes, 2009	50	14,2	0,8	58	169
3	1997	hemolytic	31/M	11,4	na	87	92	yes, 2009	50	10,2	1,1	99	87
4	1999	hemolytic	24/F	9,9	1,5	33	83	no	41	8,6	6,7	80	158
5	2002	hemolytic	20/F	8,4	6,8	80	257	yes, 2008	34	8,4	0,9	97	208
6	2002	hemolytic	44/M	11,7	2,9	99	63	yes, 2005	58	6,9	2,3	93	249
7	2004	hemolytic	68/M	9,2	9,1	60	58	no	80	13,1	5,2	6	129
8	2005	hemolytic	43/F	6,7	5,4	42	65	no	54	11,1	2,2	76	63
9	2006	hemolytic	25/M	11,5	2,5	75	210	yes, 2007	35	12,7	1,6	77	248
10	2006	hemolytic	63/F	8,1	4,0	67	59	yes, 2009	73	9	1	99	237
11	2006	hemolytic	50/M	10,8	7,8	20	79	no	60	13,9	1	13	52
12	2007	hemolytic	46/F	8,7	16,3	90	133	yes, 2008	55	11,2	1	92	125
13	2007	aplastic	31/F	9,6	1,2	14	30	no	40	10,6	4,4	75	93
14	2007	aplastic	49/F	7,9	1,9	56	48	no	58	12,1	4,7	89	71
15	2011	hemolytic	31/M	10,5	8,6	81	123	yes, 2011	36	10,5	1	94	150
16	2013	hemolytic	17/F	9,8	3,4	69	153	yes, 2014	20	12,5	1,1	66	164
17	2014	hemolytic	50/F	10,9	2,6	64	73	no	52	11,1	4,9	74	145
18	2014	hemolytic	80/F	8,1	3,1	93	115	no	82	11,1	4,0	92	124
19	2016	hemolytic	55/F	9,6	1,8	50	72	no	56	12,6	1,4	nd	77

t > ULN: times over the upper limit of normal, nd: not done, na: not available. Median values at diagnosis: WBC were 3.68 × 10^3^/mmc (range 1.7–7.04 × 10^3^/mmc); PMN 2.22 × 10^3^/mmc (0.5–3.7 × 10^3^/mmc); PLT 160 × 10^3^/mmc (38–362 × 10^3^/mmc); Median values at last follow up: WBC 3.91 × 10^3^/mmc (2.6–5.04 × 10^3^/mmc); PMN 2.24 × 10^3^/mmc (1.16–2.76 × 10^3^/mmc); PLT 159 × 10^3^/mmc (range 81–322 × 10^3^/mmc); Normal ranges: WBC 4.8–10.8 × 10^3^/mmc; PMN 1.50–6.50 × 10^3^/mmc; PLT 130–400 × 10^3^/mmc; Hb male 13.5–17.5 g/dL, Hb female 12.0–16 g/dL; LDH 135–214 U/L; Reticulocytes 20–100 × 10^3^/mmc.

Eculizumab was administered in 53% of cases because of transfusion dependence, abdominal pain and/or thrombosis. The median clone size was significantly higher in patients who underwent treatment (77.5 versus 50%, p = 0.007); likewise, the former showed a more pronounced hemolytic pattern with an increased absolute reticulocyte count (median 147 × 10^3^/mmc versus 72 × 10^3^/mmc, p = 0.007), and LDH levels (4.3 over UNL versus 2.6 folds, not significant). At enrolment the median follow-up was 10 years (range 1–20); the median hemoglobin values were increased compared to values at diagnosis, both because of treatment or transfusion support (in the eculizumab treated group from 9.2 to 11.2 g/dL and in the others from 9.6 to 11.1 g/dL). Median LDH significantly decreased in eculizumab treated cases (from 4.3 to 1 fold over ULN, p = 0.01), while it was increased (not significantly) in untreated cases (from 2.6 to 4.4 folds). Regarding the clone size, it increased from 64% to 77% (not significantly), without a different behavior in treated or untreated cases. The clone size negatively correlated with hemoglobin values (r = −0.7, p = 0.001) at the moment of the study. It is worth mentioning that subject N. 7, who had a classic transfusion-dependent hemolytic disease at diagnosis, showed a progressive amelioration overtime, with disappearance of transfusion need and remarkable reduction of GPI-negative granulocytes (from 60% to 6%), normalization of hemoglobin values (from 9.2 to 13.1 g/dL), and decrease of hemolytic parameters (LDH form 9.1 to 5.2 folds over ULN) without any therapy. On the other hand, patient N. 6 suffered from a severe hemolytic PNH with persistence of transfusion dependence on eculizumab (1200 mg every ten days).

### MRI findings

**Figure 1:**
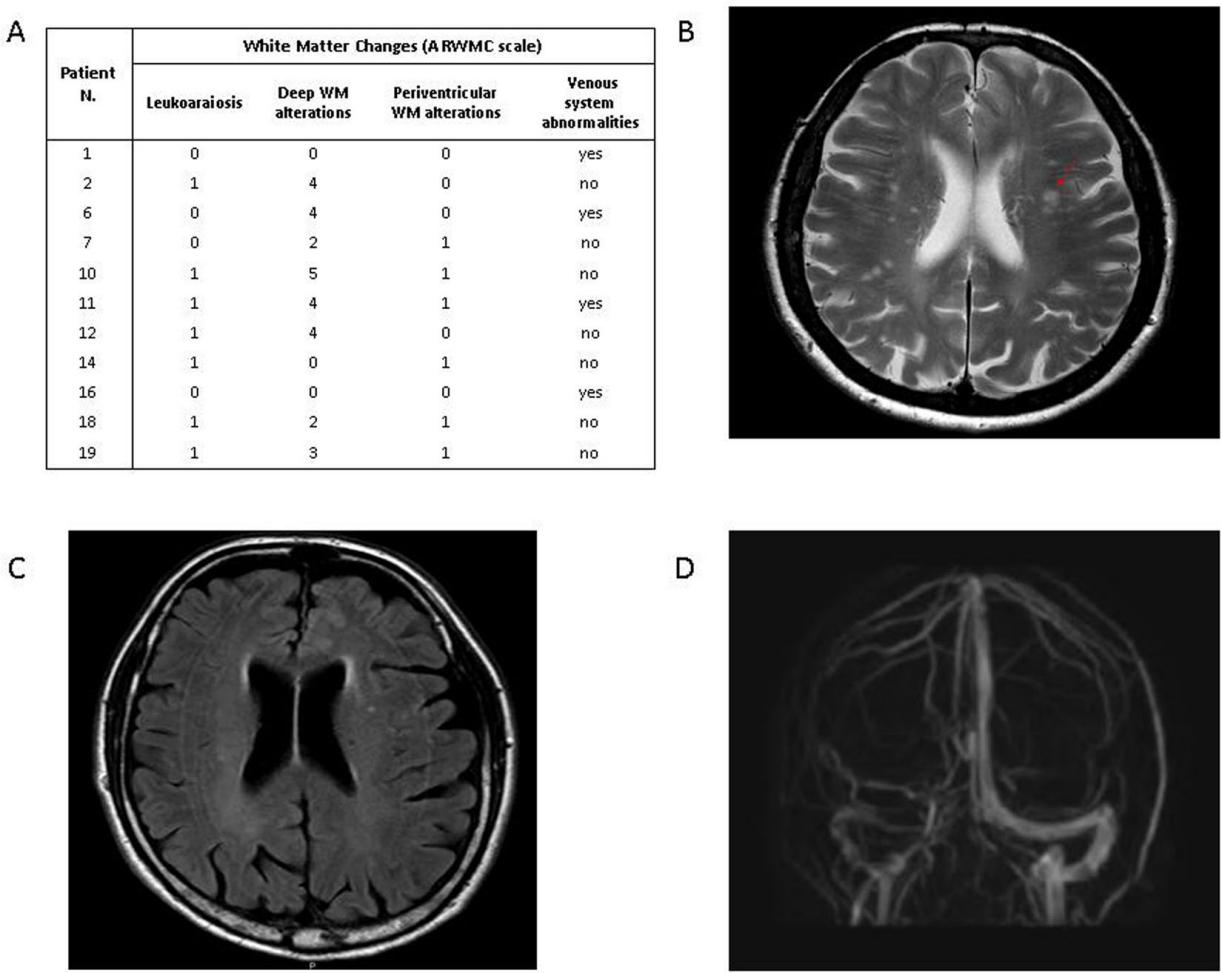
Pathological brain MRI findings in PNH patients. (**A**) White Matter Changes (ARWMC scale, see Materials and Methods) (**B**). The T2 weighted axial image shows several t2 focal hyperintensities of WM due to chronic microinfarcts (patient N.10); (**C**) The FLAIR axial image shows periventricular hyperintensity due to vascular chronic degeneration (patient N. 7); (**D**) The Phase Contrast acquisition shows the asymmetry of venous sinuses with irregular walls of the right transverse sinus that can be due to a previously occurred venous thrombosis (patient N. 1). Control population (n = 38) findings: 9 leukoaraiosis, 19 deep WM focal chronic ischemic lesions (n = 5, score 1; n = 10, score 2; n = 3, score 3; n = 1, score 4); a lesion > 5mm was observed in 2 subjects aged 53 and 83 years.

On MRI, 11/19 (58%) PNH patients showed pathological findings (Fig. 1). In particular, 9 cases displayed WM abnormalities related to chronic ischemic small vessel disease, of whom 6 periventricular WM vascular degeneration, and 8 deep WM focal chronic ischemic lesions (5 cases have both sites involved). Moreover, a focal abnormality >5 mm was detected in 2 subjects (N. 7, N. 10), and the ARWMC scale for the evaluation of WM and basal ganglia lesions gave a score >4 in 5 subjects (N. 2, N. 6, N. 10, N. 11, N. 12). No subject displayed active or previous bleeding, nor were focal alterations of the basal nuclei by the ARWMC scale found. Two patients (80 and 81 yrs) showed atrophy of the cerebral hemispheres. Regarding vascular abnormalities, one subject (N. 1) had hypoplastic left transverse sinus with irregularities in the sinus wall, suspected for prior partial venous thrombosis (Fig. 1). Three further cases (N. 6, N. 11, N. 16) displayed hypoplastic transverse sinus associated with collateral draining cortical veins, indistinguishable from anatomical variants. Intracranial artery stenosis or aneurysm, and Moya–Moya like alterations were not observed. Finally, cerebral MRI was unremarkable in 8/19 subjects. Concerning the control group, 20/38 (52.6%) subjects showed at least one pathologic finding, in particular 2 periventricular WM vascular degeneration, and 19 deep WM focal chronic ischemic lesions (1 case have both sites involved). A focal abnormality >5 mm was detected in 2 subject aged 53 and 83 years, and the ARWMC scale for the evaluation of WM and basal ganglia lesions gave a score >4 in 1 subject only (2.6%). Atrophy of the cerebral hemispheres was found in 1 subject aged 80 years. Of note, none of the controls showed vascular abnormalities, silent ischemic strokes, active or previous bleeding, or micro-hemorrhages. By comparing the two groups, the overall frequency of MRI alterations was not different between PNH and controls (58% versus 52.6%). However, the number of subjects with periventricular WM vascular degeneration was greater in the patients (6/19, 32%, versus 2/38, 5.2%, p = 0.04), along with an increased frequency of severe lesions (ARWMC scale score >4 for WM and basal ganglia lesions) (5/19, 26% versus 1/38, 2.6%, p = 0.05). Consistently, the severity of deep WM focal chronic ischemic lesions was significantly greater in PNH cases compared with controls (ARWMC scale 3.5 ± 1.07 versus 2.0 ± 0.8, mean ± SD, p < 0.0001).

The clinical and hematological parameters of patients with or without any MRI abnormality (WM and vascular alterations) are shown in Table 2. Mean age was significantly higher in the former, whereas blood counts, hemolytic markers and clone size showed no remarkable differences, except for hemoglobin values at enrolment, which were higher in cases with MRI alterations. To note, hemoglobin values at diagnosis were slightly lower and LDH greater in subjects who displayed MRI abnormalities at enrolment, and individual hemoglobin levels negatively correlated with the ARWMC score (r = −0.45, p < 0.05). No relationship was found between history of abdominal pain/thrombosis and MRI pathological findings. Likewise, MRI abnormalities were not correlated with disease duration. As regards therapy with eculizumab, a pathological MRI was found in 6/10 subjects under treatment and in 5/9 without; the sole significant vascular alteration was detected in a patient (N. 1) under treatment.

**Table 2:** Clinical and hematological parameters of patients with or without MRI abnormalities.

	***MRI Abnormalities***			
	**Yes (n** = **11)**	**No (n** = **8)**	**Yes (n** = **11)**	**No (n** = **8)**
	***Data at diagnosis***		***Data at last follow-up***	
Age (yrs)	50 ± 17*	32 ± 10	60 ± 17*	43 ± 8
Hb (g/dL)	9.5 ± 1.5	9.9 ± 1.6	12.2 ± 1.5*	10.4 ± 1.4
LDH (t > ULN)	5.8 ± 4.4	4.1 ± 2.9	2.1 ± 1.8	2.9 ± 2.2
Reticulocyte counts (x10^3^/mmc)	105 ± 60	130 ± 69	134 ± 66	148 ± 60
GPI neg PMN cells (%)	65 ± 23	60 ± 27	63 ± 34	84 ± 11
WBC (μL)	3928 ± 1260	4490 ± 1709	3702 ± 1060	3676 ± 583
PMN cells (μL)	2045 ± 961	2239 ± 885	1931 ± 655	2096 ± 471
Plt (μL)	178091 ± 86290	131875 ± 101035	165564 ± 82344	162000 ± 65937

Values are expressed as mean ± SD; *denotes p < 0.05, MRI abnormalities versus no MRI abnormalities.

### Neurological and psychiatric findings

Previous history of neurological disorders was observed in 4 patients, whereas 15 had reported no events. Patient N. 10 experienced typical transient global amnesia in his early sixties, and N. 17 seizures at five years; both these events occurred before the diagnosis of PNH. Patient N.13 complained headache since she was diagnosed with PNH. Patient N. 6 complained of progressive rest tremor in his left arm and leg twelve years after PNH diagnosis; he was diagnosed with possible Parkinson disease and started on specific treatment. Except for this subject displaying hypomimia, mild bradykinesia and rest tremor, neurological examination was unremarkable in all patients. Interestingly, patient 6 and 10 displayed significant WM MRI alterations (Fig. 1).

Eighteen subjects agreed to undergo to psychiatric assessment. Three patients resulted to have a psychiatric disorder according to SCID-I: 1 suffered from generalized anxiety disorder (GAD) (N. 4), 1 from bipolar disorder type 2 (N. 12) and 1 from adjustment disorder as a consequence of PNH diagnosis (N. 2). No patient manifested acute psychopathology at the time of assessment; however, 4 patients (N. 2, N. 4, N. 9 and N. 12) displayed mild psychiatric symptoms according to BPRS (Table 3). Only one patient (N. 7) showed a deficit of attentional and executive functions, and visual-motor coordination at TMT (prevalently in part B). As regards perception of healthy state (SF-36), 2 patients complained of remarkable limitation of work and other daily activities (RP), together with an increased perception of bodily pain (BP), and half of the patients declared an impairment of the normal social activities (SF). Surprisingly, all patients declared physical and mental well-being (no pathological findings at PF and MH) in spite of the limits imposed by their physical condition; consistently, Hb values were negatively correlated with the physical functioning scores (PF) (r = −0.43, p < 0.05), i.e. the more anaemic patients declare a fitting bodily performance. Finally, the emotional-affective well being scores (MH) negatively correlated with the disease duration (r = −0.51, p < 0.05), suggesting a progressive insight of the disease. No relationship was observed between MRI findings and neuro-psychiatric assessment.

**Table 3:** Pathological findings at psychiatric assessment.

Patient N.	BPRS	TMT-A	TMT-B	SF-36- PF	SF-36- RP	SF-36- BP	SF-36- GH	SF-36- VT	SF-36- SF	SF-36- MH
1	—	—	—	—	—	yes	—	—	—	—
2	Yes/mild	—	—	—	—	—	—	—	—	—
4	Yes/mild	—	—	—	—	yes	—	—	yes	—
7	—	yes	yes	—	yes	yes	—	—	—	—
8	—	—	—	—	—	—	—	—	yes	—
9	Yes/mild	—	—	—	—	—	—	—	yes	—
10	—	—	—	—	—	—	—	—	yes	—
11	—	—	—	—	—	—	yes	—	yes	—
12	Yes/mild	—	—	—	—	—	—	—	—	—
13	—	—	—	—	—	—	—	—	yes	—
14	—	—	—	—	—	—	yes	—	—	—
15	—	—	—	—	—	—	—	—	yes	—
17	—	—	—	—	—	—	—	—	yes	—
18	—	—	—	—	yes	yes	—	—	yes	—
19	—	—	—	—	—	—	—	yes	—	—

BPRS: Brief Psychiatry Rating Scale; TMT: Trail making test; SF-36: Short Form Health Survey; PF: Physical functioning; RP: Role-physical; BP: Bodily Pain; GH: General Health; VT: Vitality; SF: Social Functioning; MH: Mental Health. Yes indicates pathological findings. Patient N. 6 refused psychiatric assessment. Patient N. 3, N. 5, N. 16 displayed no pathological findings (not shown).

## Discussion

This is the first systematic evaluation of brain MRI findings and neuro-psychiatric status in neurologically asymptomatic PNH patients. Pathological MRI findings, involving the periventricular and deep WM as well as the vascular district, were observed in more than a half of patients, this overall frequency being not significantly different from that observed in age- and gender-matched controls. However, the frequency of severe WM lesions (both periventricular and deep) was greater in PNH, and the overall degree of ischemic lesions was significantly greater in PNH cases compared with controls. Moreover, vascular abnormalities suspected for prior partial venous thrombosis, were observed in PNH cases only. Finally, a patient showed MRI findings suggestive of prior partial thrombosis of the transverse venous sinus, consistently with the observation that the transverse sinus was the second most frequent site of cerebral venous thrombosis in PNH patients with stroke^17^.

Our results showed no definite relationship between MRI pathological findings and history of previous thrombosis/abdominal pain, nor with known parameters that identify the clinical severity of PNH, in particular the clone size and LDH. It is known that overt thrombotic events occur more frequently in patients with large clone sizes, however, they are also observed in cases with small PNH clones^22^. It is worth considering that our PNH population is mostly composed by hemolytic patients, where thrombotic complications may be more frequent. However, MRI results underline that ischemic and vascular abnormalities may be observed even in less severe cases, with smaller clones and normal hemoglobin values. Regarding therapy, of the 10 patients on eculizumab, 7 had some ischemic/vascular involvement, likely reflecting more severe and long-lasting disease. Since the study has a retrospective design and no MRI findings are available before the start of therapy, no speculations can be done on the protective effect of eculizumab on the development or worsening of vascular injury. However, it is worth mentioning that a severe focal abnormality (with a diameter greater than 5 mm) was observed in a 60 year-old man with no other known risk factors (hypertension, obesity, smoke) never treated with eculizumab. Interestingly, reduced small bowel blood flow by abdominal MRI has been useful in the decision to start therapy with eculizumab in patient N. 16^23^.

Little is known about the natural history of WM changes in the general population, starting from their development to possible subsequent progression, due to the fact that MRI imaging is expensive and impractical for large scale population based studies. In 396 patients with acute stroke followed up to 12 years, ARWMC seems to be a significant predictor of poor long term survival and death by ischemic stroke^24^. Moreover, in a large non-institutionalized cohort of subjects aged >65 years without a history of stroke and followed-up for more than 10 years, ARWMCs predicted total cardiovascular and non-cardiovascular mortality^25^. MRI is considered the gold standard to identify both ischemic and vascular lesions of central nervous system and it is increasingly used in hemolytic disorders with known ischemic involvement, such as sickle cell disease (SCD), thrombotic microangiopathies and vasculitis^26–29^. In SCD up to 40% of cases were found to display silent cerebral infarction by MRI and these changes are related to cognitive impairment in adults^26,30^ and seem to start early in the course of the disease^31^. More recently, by resting state functional MRI, a selective disruption of connectivity among relevant regions of the brain was observed in SCD, potentially leading to reduced cognition and altered functional brain dynamics^32^. In our cases, no significant cognitive impairment was found despite MRI alterations. Neither significant psychiatric symptoms nor a perception of poor health were found, although PNH subjects had insight about limitations in social functioning. Paradoxically, patients with more severe forms of illness (e. g, with lower hemoglobin values) reported higher levels of physical activities. One explanation, in absence of acute psychiatric disorders, might be that patients can benefit of a specialized care, and this may affect patients’ mood and related energies. Whether these observations are characteristic of PNH or are related to a chronic disease is difficult to define and may deserve further investigation.

In conclusion, brain MRI was able to reveal chronic ischemic and vascular lesions in a significant proportion of neurologically asymptomatic PNH patients, similarly to what reported in SCD, suggesting that these hemolytic conditions may share a common thrombophlic diathesis. As for abdominal MRI, brain evaluation may add further information on the severity of a specific PNH patient, and be useful in the decision to start therapy, which is not always easy on the clinical/hematological basis. Although WM changes are largely related to age, our findings suggest being aware of possible asymptomatic vaso-occlusive events in PNH, leading to chronic small vessel disease in the brain, even in absence of neurological symptoms. This is of particular importance for younger patients, although only an *ad hoc* follow-up with MRI at diagnosis and at 2–3 years intervals will disclose whether or not these lesions would progress, remain stable, or regress, or may be influenced by therapy with eculizumab.

## Patients and Methods

### Patients

19 consecutive patients diagnosed with PNH since 1996 till 2015 and followed at our Institution were enrolled from June 2015 until May 2016. The study was approved by the Ethical Committee of Human Experimentation of Area 2 Milano. All patients gave informed consent and the study has been conducted in accordance with the Declaration of Helsinki and with the EU ICH GCP Guidelines. Clinical and hematologic parameters at diagnosis, at enrolment and along the follow up were evaluated. PNH was diagnosed by cytometric analysis of CD55 and CD59 deficient granulocytes, monocytes, and erythrocytes by the fluorescent aerolysin (FLAER) method, and classified in the known categories: classical hemolytic, PNH in the context of other primary bone marrow disorders, such as aplastic anemia or myelodysplastic syndrome, and subclinical PNH, in which patients have small PNH clones but no clinical or laboratory evidence of hemolysis or thrombosis^5^. None of the subjects studied had previous history of stroke, head trauma, neurosurgery, and overt neurological disease, or reported known hereditary or acquired thrombophilic states.

### MRI evaluation

All patients underwent non-enhanced cerebral magnetic resonance imaging (MRI), and intracranial arterial and venous MR-angiography (MRA). Brain MRI scans were obtained using a 3.0-T scanner (Achieva, Philips Medical Systems). As controls we evaluated 38 consecutive age and sex-matched subjects (median age 44.5 years, range 23–87, 22 female and 16 male) who underwent MRI for reasons unrelated to ischemic symptoms (i.e. otalgia, ocular pain or sella turcica study), and showed no major pathological findings. Moreover, none of the controls had prior diagnosis of venous or arterial cerebral thrombosis, previous history of head trauma or neurosurgery. The following sequences were acquired from all subjects: (1) T2-weighted turbo spin echo (TSE) (repetition time [TR] 2491 ms; echo time [TE] 77 ms; flip angle 90°; 4-mm-thick axial slices; matrix size 505 × 512; field of view [FOV] 230 × 230 mm^2^); (2) fluid-attenuated inversion recovery (FLAIR) (TR 11.000 ms; TE 125 ms; flip angle 90°; 4-mm-thick axial slices; matrix size 249 × 344; FOV 230 × 230 mm^2^); (3) 3D T1- weighted fast field echo (TR 9.8 ms, TE 4.6 ms, flip angle 8°, FOV 240 × 240 mm^2^, matrix 240 × 240, slice thickness 1 mm, 220 contiguous sagittal slices); and (4) T2-weighted fast field echo (FFE) (TR 839 ms; TE 16 ms; FA 18°; 4-mm-thick axial slices; matrix size 205 × 256; FOV 230 × 230 mm2); (5) diffusion Echo Planar (TR 3367 ms; TE 52 ms; FA 90°; 4 mm-thick-axial slice; matrix size 124 × 124; FOV 250 × 250 mm2; b-values 0–1000 s/mm^2^); (6) 3D Time of Flight MRA (TR 25 ms; TE 3,4 ms; FA 20°; 1-mm-thick-contiguous axial size; matrix size 407 × 800; 200 × 200mm^2^); (7) Phase Contrast MRA (TR 17 ms; TE 6 ms; FA 10°; 1.6-mm-thick- contiguous axial slices; matrix size 195 × 256; FOV 230 × 230 ms; velocity encoding 15 cm/s.

The following variables were evaluated by two blinded independent observers: a) chronic ischemic small vessel disease defined as WM abnormalities with T2 and FLAIR hyperintense signal and quantified according to age related white matter changes (ARWMC) scale^33^ with distinction between deep white matter (WM) and basal ganglia (deep grey matter); b) diffuse T2 and FLAIR signal hyperintensity of the periventricular WM, consistent with chronic vascular degeneration or leukoaraiosis; c) focal alterations consistent with old silent ischemic strokes, defined as a malacic fluid-filled cavity (signal similar to cerebrospinal fluid, CSF) presenting with hyperintense signal on T2-weighted images and hypointense signal on FLAIR images without a history or physical findings of a focal neurological deficit^34^; d) active or previous bleeding or micro-hemorrhages; e) atrophy. Moreover, abnormalities of the circle of Willis and its branches (stenosis of intracranial arteries, aneurysms, Moya–Moya like alterations), and of cerebral venous sinus were evaluated. For WM lesions, the following scores were calculated: 0 = no lesions, 1 = focal lesion, 2 = initial confluence of the lesions, 3 = broad involvement of the entire region with or without involvement of the U fibers. Likewise, for basal ganglia, 0 was used in case of no lesions, 1 for one focal lesion, 2 for more than one focal lesion, and 3 for confluent lesions. Scores were calculated separately for periventricular and deep WM, and evaluated separately for each side in the frontal, parietal-occipital and temporal lobes, in the infratentorial compartment, and in the basal ganglia. Furthermore, a more global score for leukoaraiosis was attributed 0 = absent, 1 = mild to moderate, and 2 = severe.

### Neuro-psychiatric evaluation

Psychiatric evaluation included the following scales: the brief psychiatric rating scale (BPRS), the structured clinical interview for axis I DSM-IV-TR disorders (SCID-1)^35,36^, the short form (SF-36) health survey, and the trail making test (TMT). BPRS was used to measure general psychopathology, and total scores higher than 31 were considered pathological according to previous literature^37^. SF-36 was aimed to assess patients’ disability in seven different areas (physical activity, physical health, pain, general health, vitality, social functioning, mental health); pathological findings were defined according to a large Italian sample^38^. TMT (part A and B) was used to assess patients’ cognitive functioning and specifically attention and executive functioning; a time of execution >78 seconds for TMT-A and >273 seconds for TMT-B were considered as abnormal^39^. Of note, SF-36 has already been used to assess disability of hematological patients^40^. Psychiatric evaluations were performed by the same trained rater. A complete clinical neurologic assessment was also performed.

### Statistical analysis

Descriptive analyses were performed on the total sample. Chi-square tests were used to compare qualitative variables, while Student’s t tests to compare continuous ones. Finally, Pearson’s linear correlations were performed to relate clinical features of the total sample.

## References

[1] Hill A, DeZern AE, Kinoshita T, Brodsky RA (2017). Paroxysmal nocturnal haemoglobinuria. Nat Rev Dis Primers.

[2] Hill A, Kelly RJ, Hillmen P (2013). Thrombosis in paroxysmal nocturnal haemoglobinuria. Blood.

[3] Kozuma Y (2011). Procoagulant properties of microparticles released from red blood cells in paroxysmal nocturnal haemoglobinuria. Br J Haematol.

[4] Brodsky, R. A. Complement in haemolytic anaemia. *Bloo*d *(Haematology American Society of Haematology*, *Educational Program* 385–391, 10.1182/asheducation-2015.1.385 (2015).

[5] de Latour RP (2008). French Society of Hematology; French Association of Young Hematologists. Paroxysmal nocturnal haemoglobinuria: natural history of disease subcategories. Blood.

[6] Schrezenmeier H (2014). Baseline characteristics and disease burden in patients in the International Paroxysmal Nocturnal Haemoglobinuria Registry. Haematologica.

[7] Luzzatto L. Recent advances in the pathogenesis and treatment of paroxysmal nocturnal hemoglobinuria. *F1000Res* **5**, 10.12688/f1000research.7288.1 (eCollection 2016). 10.12688/f1000research.7288.1 PMC4765720 26962442

[8] Hillmen P (2006). The complement inhibitor eculizumab in paroxysmal nocturnal hemoglobinuria. New EngL J Med.

[9] Hillmen P (2007). Effect of the complement inhibitor eculizumab on thromboembolism in patients with paroxysmal nocturnal hemoglobinuria. Blood.

[10] Brodsky RA (2008). Multicenter phase 3 study of the complement inhibitor eculizumab for the treatment of patients with paroxysmal nocturnal hemoglobinuria. Blood.

[11] Hill A (2010). Effect of eculizumab on haemolysis-associated nitric oxide depletion, dyspnoea, and measures of pulmonary hypertension in patients with paroxysmal nocturnal haemoglobinuria. Br J Haematol.

[12] Kelly RJ (2011). Long-term treatment with eculizumab in paroxysmal nocturnal haemoglobinuria: sustained efficacy and improved survival. Blood.

[13] Loschi M (2016). Impact of eculizumab treatment on paroxysmal nocturnal haemoglobinuria: a treatment versus no-treatment study. Am J Hematol.

[14] Samadder NJ, Casaubon L, Silver F, Cavalcanti R (2007). Neurological complications of paroxysmal nocturnal haemoglobinuria. Can J Neurol Sci.

[15] Memon AR, Khan R, Rauf MU, Shafique K (2014). Paroxysmal nocturnal haemoglobinuria presenting as cerebral venous sinus thrombosis: a case report. Int Arch Med.

[16] Shrestha GS (2016). Cerebral venous thrombosis presenting with intracerebral haemorrhage in a patient with paroxysmal nocturnal haemoglobinuria. Indian J Crit Care Med.

[17] Meppiel E (2015). Cerebral Venous Thrombosis in Paroxysmal Nocturnal Haemoglobinuria A Series of 15 Cases and Review of the Literature. Medicine (Baltimore).

[18] Audebert HJ (2005). Cerebral ischemic infarction in paroxysmal nocturnal haemoglobinuria report of 2 cases and updated review of 7 previously published patients. J Neurol.

[19] Meira AT (2017). Multiple Lacunar Infarcts in Paroxysmal Nocturnal Hemoglobinuria. J Stroke Cerebrovasc Dis.

[20] Mugikura S (2010). Unilateral moyamoya syndrome involving the ipsilateral anterior and posterior circulation associated with paroxysmal nocturnal haemoglobinuria. Jpn J Radiol.

[21] Yen TA (2012). Eculizumab treatment of paroxysmal nocturnal haemoglobinuria presenting as moyamoya syndrome in a 9-year-old male. Pediat Blood Cancer.

[22] Lee JW (2013). Clinical signs and symptoms associated with increased risk for thrombosis in patients with paroxysmal nocturnal haemoglobinuria from a Korean Registry. Int J Haematol.

[23] De Cobelli, F. *et al*. New Insights in Abdominal Pain in Paroxysmal Nocturnal Haemoglobinuria (PNH): A MRI Study. *PLoS One*, **10**, e0122832, 10.1371/journal.pone.0122832 (ecollection 2015). 10.1371/journal.pone.0122832 PMC4405271 25897796

[24] Oksala NK (2009). Age related white matter changes predict stroke death in long term follow-up. J Neurol, Neurosur, and psychiatry.

[25] Kuller LH (2007). White matter grade and ventricular volume on brain MRI as markers of longevity in the cardiovascular health study. Neurobiol Aging.

[26] Quinn, C.T. Breakthrough: new guidance for silent cerebral ischemia and infarction in sickle cell disease. *Blood (American Society of Haematology Educational Program)*, 438–443, 10.1182/asheducation-2014.1.438 (2014). 10.1182/asheducation-2014.1.438 25696891

[27] Weissenborn K (2013). Quantitative MRI shows cerebral microstructural damage in hemolytic-uremic syndrome patients with severe neurological symptoms but no changes in conventional MRI. Neuroradiol.

[28] Abe G, Kikuchi H, Arinuma Y, Hirohata S (2016). Brain MRI in patients with acute confusional state of diffuse psychiatric/neuropsychological syndromes in systemic lupus erythematosus. Mod Rheumatol.

[29] González-Suárez. I, Arpa J, Ríos-Blanco JJ (2016). Brain Microvasculature Involvement in ANCA Positive Vasculitis. Cerebrovasc Dis.

[30] Mackin RS (2014). Neuroimaging abnormalities in adults with sickle cell anemia: associations with cognition. Neurology.

[31] Manara R (2017). Longitudinal evaluation of cerebral white matter hyperintensities lesion volume in children with sickle cell disease. Br J Haematol.

[32] Colombatti, R. *et al*. Cognition and the Default Mode Network in Children with Sickle Cell Disease: A Resting State Functional MRI Study. *PLoS One*, **11**, e0157090, 10.1371/journal.pone.0157090 (ecollection 2016). 10.1371/journal.pone.0157090 PMC4900543 27281287

[33] Wahlund LO (2001). European Task Force on Age-Related White Matter Changes. A new rating scale for age-related white matter changes applicable to MRI and CT. Stroke.

[34] Wardlaw JM (2013). STandards for ReportIng Vascular changes on nEuroimaging (STRIVEv1). Neuroimaging standards for research into small vessel disease and its contribution to ageing and neurodegeneration. Lancet Neurol.

[35] American Psychiatric Association (APA). Diagnostic and Statistical Manual of Mental Disorders, 4th edition, text revised. (American Psychiatric Press: Washington D.C. 2000).

[36] First, M.B., Spitzer, R.L., Gibbon, M. & Williams, J.B.W. Structured Clinical Interview for DSM-IV-TR Axis I Disorders, Research Version, Patient Edition. (SCID-I/P). (Biometrics Research, New York State Psychiatric Institute (2002).

[37] Leucht S (2005). Clinical implications of Brief Psychiatric Rating Scale scores. Br J Psychiatry.

[38] Apolone G, Mosconi P (1998). The Italian SF-36 Health Survey: translation, validation and norming. J Clin Epidemiol.

[39] Goul WR, Brown M (1970). Effects of age and intelligence on trail making test performance and validity. Percep Mot Skills.

[40] Zhou Z (2007). Health-related quality of life measured by the Short Form 36 in immune thrombocytopenic purpura: a cross-sectional survey in China. Eur J Hematol.

